# A simulator based on the morphology of an actual stomach for training in endoscopic therapy: is this where beginners should be starting?

**DOI:** 10.1055/a-2088-0213

**Published:** 2023-05-26

**Authors:** Jiyu Zhang, Miao Shi, Saif Ullah, Lixia Zhao, Bing-Rong Liu

**Affiliations:** Division of Gastroenterology, The First Affiliated Hospital of Zhengzhou University, Henan Province, P. R. China

At present, the design of most endoscopic training simulators is not based on actual gastric morphology 1 2 3 . We therefore developed a simulator for endoscopic therapy training that was based on the morphology of an actual stomach. Starting from computed tomographic images of a stomach, a standard gastric morphological structure was made using computer modeling and 3 D printing technology ( Fig. 1 a–c ). To simulate the position of the human stomach during gastroscopy, a container was designed to support and hold the simulator in a fixed position ( Fig. 1 d ). Compared to traditional simulators, which are not designed on the basis of real gastric morphology, our simulator preserves the original structure of the stomach as much as possible, especially in regard to the fundus and the angular incisure ( Fig. 2 a ). This allows trainees to experience clinical gastroscopy in a way that is as realistic as possible.

**Fig. 1 a:**
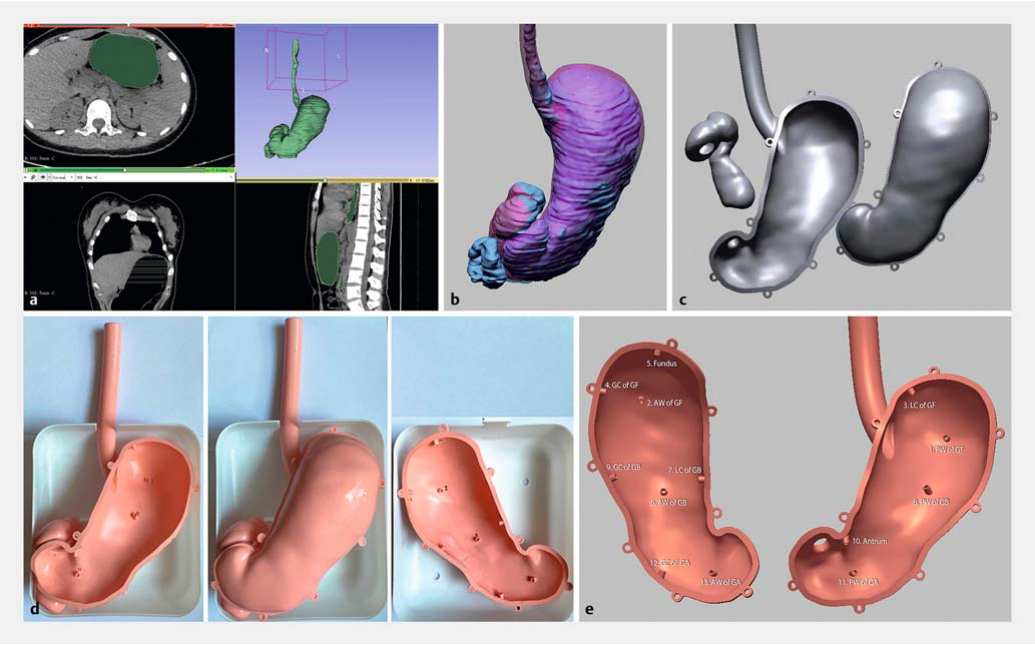
CT imaging produces a basic gastric morphological structure. **b** The prototype structure is optimized. **c** The 3D-printed simulator is optimized and divided into three parts. **d** The assembled training model in its container. **e** Sequentially numbered hollow cylinders with labels marking standard locations in the stomach, e. g., 1. PW of GF: no. 1, posterior wall of the gastric fundus.

**Fig. 2 a:**
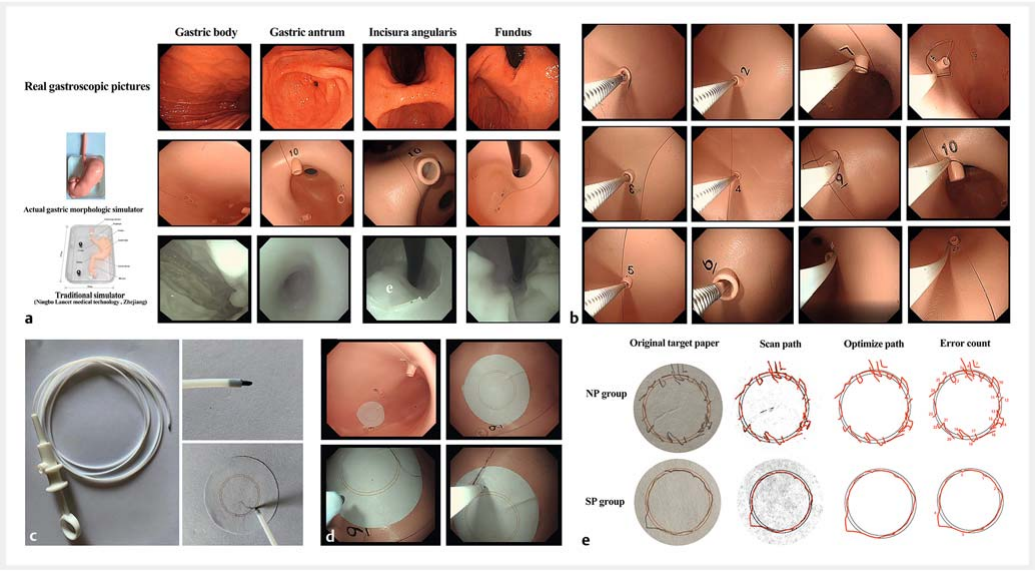
Comparison between real gastroscopy (top row), the new gastric morphology simulator based on an actual stomach (middle row), and a traditional simulator (bottom row). **b** Gastroscopic views for training in precise insertion and simulated snare polypectomy. **c** Adhesive target paper and writing pen. **d** Gastroscopic view for simulated endoscopic submucosal dissection procedure. **e** Computer processing of the training results.

Accurate control of the endoscope is the most important skill for performing various endoscopic procedures. Therefore, two kinds of training modules have been designed, which use, respectively, (1) sequentially numbered hollow cylinders (nos. 1–13) ( Fig. 1 e ), and (2) adhesive target paper and a writing pen. The hollow cylinders are distributed over the inner wall of the simulator, each representing a standard gastric location, e. g., no. 1 for the posterior wall of the gastric fundus. As the orifices of the hollow cylinders are perpendicular to the gastric wall with which they are in contact, their angles of inclination vary. The following functions are served:

(1) Accurate indication of the various locations in the gastric anatomical structure. The trainees can determine the correct gastric anatomical location of the observed part in the endoscopic field according to the numbers observed during training, which helps them to master the anatomical morphology of a real stomach.

(2) Training in specialized endoscopic skills with different instruments, such as precise insertion or simulated snare polypectomy ( Fig. 2 b ).

The adhesive target paper can be pasted onto the different locations in the simulator to give training in the use of the endoscope in various difficult anatomical sites. The writing pen is designed to work with the paper. The head of the pen is connected with a 1.2-m hollow soft plastic tube and can pass through the biopsy channel of the endoscope. This allows the simulated performance of an endoscopic submucosal resection procedure ( Fig. 2 c, d ). After the training, the target paper is taken out and the computer is used to convert the lines drawn into vector route records and perform data analysis, which can reflect the operating level in an intuitive way ( Fig. 2 e ; Video 1 ).

**Video 1** A simulator based on actual gastric morphology for training in endoscopic therapy.


In conclusion, this simulator of real gastric morphology for endoscopic therapy training is re-usable, convenient, and authentic. It is very helpful in training precision control of the endoscope in a realistic training situation using the training modules provided. It can also be used as a standard test tool for measuring operators’ level of skill.

Endoscopy_UCTN_Code_TTT_1AU_2AB

## Notes

Endoscopy E-Videos

https://eref.thieme.de/e-videos
*E-Videos* is an open access online section of the journal *Endoscopy* , reporting on interesting cases and new techniques in gastroenterological endoscopy. All papers include a high-quality video and are published with a Creative Commons CC-BY license. Endoscopy E-Videos qualify for HINARI discounts and waivers and eligibility is automatically checked during the submission process. We grant 100% waivers to articles whose corresponding authors are based in Group A countries and 50% waivers to those who are based in Group B countries as classified by Research4Life (see: https://www.research4life.org/access/eligibility/ ). This section has its own submission website at https://mc.manuscriptcentral.com/e-videos
